# Discharged Officers and Their Outlook

**Published:** 1919-01-18

**Authors:** W. Smith

**Affiliations:** "A Hospital Committeeman." 15 Victoria Park, Manchester


### "Discharged Officers and their Outlook."

*To the Editor of* The Hospital.

Sir,—Further to my letter and my comments thereon, I would be glad if you would kindly publish the following: I stoutly assert that the best and only man for a secretaryship is he who has had years of experience in hospitals, who possesses initiative, business acumen, energy, tact, and ability to deal in a highly satisfactory manner with the many conditions and problems which are peculiar to such institutions.

As I stated in my previous communication, I have seen the folly of appointing as secretary a man of "education" and experience other than in a hospital, for in a short time by reason of being untrained he involved the institution in a loss of hundreds of pounds by failure to obtain income and in purchasing commodities.

Never was it so imperative as in these days of financial stress, acute problems generally, and changing circumstances, that only men of hospital experience and undoubted ability be chosen for high administrative office. I repeat that to appoint other than such is to do a hospital disservice and to fail in the performance of a great duty.—Yours faithfully, W. Smith.

"A Hospital Committeeman."

15 Victoria Park, Manchester, January 5, 1919.

[No man is born with hospital experience; he must begin at some time. A well-educated, capable business man will soon learn. Such men can be found amongst wounded officers.—Ed. The Hospital.]

